# Expression of miR-34a in T-Cells Infected by Human T-Lymphotropic Virus 1

**DOI:** 10.3389/fmicb.2018.00832

**Published:** 2018-05-04

**Authors:** Varun K. Sharma, Vittoria Raimondi, Katia Ruggero, Cynthia A. Pise-Masison, Ilaria Cavallari, Micol Silic-Benussi, Vincenzo Ciminale, Donna M. D’Agostino

**Affiliations:** ^1^Department of Surgery, Oncology and Gastroenterology, University of Padova, Padova, Italy; ^2^Veneto Institute of Oncology IOV – IRCCS, Padova, Italy; ^3^Animal Models and Retroviral Vaccines Section, National Cancer Institute, National Institutes of Health, Bethesda, MD, United States; ^4^Department of Biomedical Sciences, University of Padova, Padova, Italy

**Keywords:** HTLV-1, miR-34a, p53, nutlin-3a, adult T-cell leukemia/lymphoma

## Abstract

Human T-lymphotropic virus 1 (HTLV-1) immortalizes T-cells and is the causative agent of adult T-cell leukemia/lymphoma (ATLL). HTLV-1 replication and transformation are governed by multiple interactions between viral regulatory proteins and host cell factors that remain to be fully elucidated. The present study investigated the impact of HTLV-1 infection on the expression of miR-34a, a microRNA whose expression is downregulated in many types of cancer. Results of RT-PCR assays showed that five out of six HTLV-1-positive cell lines expressed higher levels of miR-34a compared to normal PBMC or purified CD4+ T-cells. ATLL cell line ED, which did not express miR-34a, showed methylation of the miR-34a promoter. Newly infected PBMC and samples from 10 ATLL patients also showed a prominent increase in miR-34a expression compared to PBMC controls. The primary miR-34a transcript expressed in infected cell line C91PL contained binding motifs for NF-κB and p53. Pharmacological inhibition of NF-κB with Bay 11-7082 indicated that this pathway contributes to sustain miR-34a levels in infected cells. Treatment of infected cell lines with the p53 activator nutlin-3a resulted in a further increase in miR-34a levels, thus confirming it as a transcriptional target of p53. Nutlin-3a-treated cells showed downregulation of known miR-34a targets including the deacetylase SIRT1, which was accompanied by increased acetylation of p53, a substrate of SIRT1. Transfection of C91PL cells with a miR-34a mimic also led to downregulation of mRNA targets including SIRT1 as well as the pro-apoptotic factor BAX. Unlike nutlin-3a, the miR-34a mimic did not cause cell cycle arrest or reduce cell viability. On the other hand, sequestration of miR-34a with a sponge construct resulted in an increase in death of C91PL cells. These findings provide evidence for a functional role for miR-34a in fine-tuning the expression of target genes that influence the turnover of HTLV-1-infected cells.

## Introduction

Human T-lymphotropic virus 1 (HTLV-1) infects approximately 5–10 million people worldwide (Gessain and Cassar, 2012). About 5% of infected individuals develop an aggressive malignancy of mature CD4+ T-cells termed adult T-cell leukemia/lymphoma (ATLL) or a progressive demyelinating neurological disease termed tropical spastic paraparesis/HTLV-associated myelopathy (TSP/HAM) (Poiesz et al., 1980; Yoshida et al., 1982; Gessain et al., 1985; Osame et al., 1986). The transforming potential of HTLV-1 is attributable primarily to the viral proteins Tax (reviewed by Currer et al., 2012) and HBZ (reviewed by Ma et al., 2016), which are each capable of inducing hematological malignancies when expressed as transgenes in mice (Hasegawa et al., 2006; Satou et al., 2011). However, the long clinical latency and low penetrance of ATLL suggest that other viral and cellular factors contribute to determine the fate of HTLV-1-infected cells (for a recent review on HTLV-1, see Bangham, 2017).

The emerging importance of microRNAs (miRNAs) as fine-tuners of gene expression has prompted studies of the interplay between HTLV-1 and the miRNA network. The first such study showed that HTLV-1-infected cell lines express high levels of miR-21, miR-24, miR-146a, and miR-155 and reduced levels of miR-223 compared to normal CD4+ T-cells and uninfected T-cell lines (Pichler et al., 2008). ATLL cells also exhibit important differences in miRNA expression compared to normal peripheral blood mononuclear cells (PBMC) or CD4+ T-cells (Yeung et al., 2008; Bellon et al., 2009; Yamagishi et al., 2012). The first miRNA profiling study of ATLL samples revealed elevated expression of miR-93 and miR-130b, which target the mRNA coding for the pro-apoptotic protein TP53INP1 (Yeung et al., 2008). An analysis of a large panel of ATLL samples revealed an overall downregulation of miRNAs, including miR-31, an important target of which is NIK, an activator of the NF-κB pathway (Yamagishi et al., 2012). miR-145 is downregulated in HTLV-1-positive cell lines and ATLL samples, a property that correlates with poor ATLL patient survival; furthermore, miR-145 has growth suppressive effects when expressed in an ATLL cell line (Xia et al., 2014). Downregulation of miR-150 and miR-223 in HTLV-1-transformed cells results in loss of control of their target STAT1 (Moles et al., 2015). The viral proteins Tax, Rex, and HBZ interfere with the production of mature miRNAs by promoting degradation of Drosha (Tax; Van Duyne et al., 2012) and by inhibiting Dicer expression (HBZ; Gazon et al., 2016) and Dicer activity (Rex; Abe et al., 2010). On the other hand, Tax can upregulate expression of miR-130b (Yeung et al., 2008), miR-146a (Pichler et al., 2008; Tomita et al., 2009), and miR-155 (Tomita, 2012); TRAF6, an adaptor protein involved in a variety of signal transduction pathways, was identified as a target of miR-146a in HTLV-1-infected cell lines (Tomita et al., 2009). HBZ upregulates miR-17 and miR-21, which target proteins that control chromatin remodeling (Vernin et al., 2014). Binding of the HTLV-1 genomic RNA by miR-28-3p interferes with reverse transcription, thereby blocking productive infection (Bai and Nicot, 2015).

In a previous analysis of small RNA libraries, we observed that miR-34a, a highly conserved miRNA that is a component of the p53 pathway and is downregulated in many types of cancer (reviewed by Hermeking, 2010; Slabakova et al., 2017), was more abundant in HTLV-1-infected cell lines C91PL and MT-2 compared to control CD4+ cells (Ruggero et al., 2014). As described below, further investigation of the expression of miR-34a confirmed its upregulation in the context of HTLV-1 infection and identified targets with key roles in cell survival and death.

## Materials and Methods

### Cell Culture

HTLV-1-positive T-cell lines HUT-102 (Poiesz et al., 1980), C91PL (Popovic et al., 1983), MT-2 (Popovic et al., 1983), C8166 (Bhat et al., 1993), ATL-2 (Maeda et al., 1985), and ED-40515(–) [an IL-2-independent subclone (Arima et al., 1992) of ED-40515 (Maeda et al., 1985), referred to as ED in the present study] and the T-ALL cell line Jurkat were maintained in RPMI-1640 (Sigma-Aldrich or Euroclone) supplemented with 10% fetal bovine serum (FBS, Invitrogen), 100 units/mL penicillin and 20 units/mL streptomycin (Euroclone, complete RPMI). HeLa cells were maintained in Dulbecco’s Modified Eagles Medium (DMEM, Sigma-Aldrich or Euroclone) supplemented with 10% FBS and penicillin/streptomycin. Genetic profiling was carried out on the cell lines with the PowerPlex 16 HS kit (Promega, Raimondi et al., 2017). CD4+ cells were isolated from normal peripheral blood mononuclear cells (PBMC) using the MACS CD4+ T cell Isolation Kit II or MACS CD4 Microbeads (Miltenyi Biotec). The resulting preparations contained ≥90% CD4+ cells measured by flow cytometry using a PE-labeled anti-CD4 antibody (Becton Dickinson). PBMC samples from ATLL patients (described in Pise-Masison et al., 2009) were collected in the context of National Cancer Institute Institutional Review Board-approved studies (IRB nos. 00-C-0030 and 03-C-0194) with informed consent obtained from all subjects in accordance with the Declaration of Helsinki.

### Infection of PBMC

C91PL cells were lethally irradiated (69.5 Gy). PMBC from two blood donors were cultured for 3 h and monocytes/macrophages were depleted by adhesion. 2×10^6^ irradiated C91PL cells were cultured together with 2.5×10^6^ PBMC in a total volume of 4.5 mL complete RPMI supplemented with 500 μg/mL phytohemagglutinin (PHA, Sigma-Aldrich). Control cultures containing only irradiated C91PL cells or PBMC stimulated with PHA were set up in parallel. After 2 days, cultures were supplemented interleukin-2 (IL-2, 20 U/mL, Roche). The control irradiated C91PL cells were dead after 2 weeks. RNA was isolated from aliquots of the stimulated PBMC after 8 days’ culture and from aliquots of the co-cultures at days 30 and 72. Genomic DNA was isolated at the 72-day time point using a salting-out method (Qiagen Flexigene kit) and analyzed by PCR with primers specific for the HTLV-1 U5-gag region (Ruggero et al., 2014); PCR with primers specific for the pri/pre-miR-34a region (see below) served as a control. PCR products were separated in non-denaturing polyacrylamide gels, stained with GelRed (Biotium) and photographed using a Cambridge UVTEC system.

### RNA Isolation

Total RNA was isolated using TRIZol (Life Technologies) or TriReagent (Sigma-Aldrich) according to the manufacturer’s protocol and quantified using a Nanodrop or Implen spectrophotometer.

### Sequencing of the TP53 Open Reading Frame

Total RNA was reverse-transcribed using the Superscript VILO cDNA Synthesis Kit (Invitrogen) according to the manufacturer’s protocol. The resulting cDNA was PCR-amplified using TP53-specific primers described elsewhere (Muscolini et al., 2008). Amplicons were subjected to Sanger sequencing (Bigdye kit, Applied Biosystems) and analyzed on a 3730xl DNA Analyzer (Applied Biosystems). The resulting sequences were compared to NCBI p53 transcript reference sequence NM_000546.5 using Finch TV Version 1.4.0 (Geospiza).

### miR-34a Promoter Methylation Analysis

Genomic DNA (500 ng) was subjected to bisulfite conversion, desulfonation and column-purification (EZ DNA Methylation-Gold kit, Zymo Research). One-tenth of the eluted DNA was PCR-amplified using primers specific for methylated and unmethylated CpG sites in a 170-bp segment spanning chr1 nt 9182497-9182328 (complementary strand; Chim et al., 2010). PCR products were separated by non-denaturing PAGE in 2% agarose gels. The efficiency of bisulfite conversion was evaluated by subjecting the methylated-MSP product obtained for cell line ED to Sanger sequencing as described above.

### RACE

Total RNA isolated from C91PL cells was subjected to RACE (rapid identification of cDNA ends) using the FirstChoice RLM RACE kit (Ambion) according to the manufacturer’s instructions. The 5′RACE was carried out using outer primer 5′RACE P1 (AGAGCTTCCGAAGTCCTGG) and inner primer 5′RACE P2 (TTGCTCACAACAACCAGCTAAGA) described by Navarro et al. (Navarro et al., 2009). 3′RACE was performed using outer primer 3′RACE P1 (GGACTTCGGAAGCTCTTCTG) and inner primer 3′RACE P2 (TGGGAAAGTGAGCTCCAGG). Second-round PCR products were separated on non-denaturing acrylamide gels. The most abundant product was eluted and sequenced using primer 5′RACE P2 or 3′RACE P2.

### Quantitative RT-PCR

Total RNA was subjected to reverse transcription and qPCR using Taqman microRNA assays (Applied Biosystems) and a 7900HT Fast (Applied Biosystems) or LightCycler 480 (Roche) thermal cycler. RNU44 served as an endogenous control. Results were analyzed by relative quantification using the 2^-ΔΔCT^ method and a calibrator specified in the figure legends. A threshold cycle (C_T_) of 35 was considered “undetermined.” For mRNA expression analysis, RNA was treated with DNase I (Invitrogen) and then reverse-transcribed using SuperScript II reverse transcriptase (Invitrogen) and random hexamers (Applied Biosystems) (**Figures 5**, **7** and Supplementary Figures 1, 4) or with the Superscript VILO cDNA Synthesis Kit (without DNAse treatment) (Supplementary Figure 3). cDNA was PCR-amplified by using a SYBR Green master mix (Roche or Thermo Scientific) and the following primer pairs: CDKN1A-s (AGACTCTCAGGGTCGAAAAC) and CDKN1A-as (TTCCAGGACTGCAGGCTTC); pri/pre-miR-34a-s (TGGCAGTGTCTTAGCTGGTTG) and pri/pre-miR-34a-as (GGCAGTATACTTGCTGATTGCTT) (Jiang et al., 2005); TP53INP1-s (CTTCCTCCAACCAAGAACCAG) and TP53INP1-as (CAAGCACTCAAGAGATGCCG); BAX-s (GTCTTTTTCCGAGTGGCAGC) and BAX-as (AGGAAGTCCAATGTCCAGCC); BIRC5-s (TTCTCAAGGACCACCGCATC) and BIRC5-as (TGAAGCAGAAGAAACACTGGG); SIRT1-s (ACATAGACACGCTGGAACAGG) and SIRT1-as (GATAGCAAGCGGTTCATCAGC); TP53-s (TGGAAGGAAATTTGCGTGTGG) and TP53-as (CCAGTGTGATGATGGTGAGG); ACTB-s (AGCACAGAGCCTCGCCTTTG) and ACTB-as (GGAATCCTTCTGACCCATGC); B2M-s (TGACTTTGTCACAGCCCAAG) and B2M-as (TTCAAACCTCCATGATGCTG); GAPDH-s (GAAGGTGAAGGTCGGAGTC) and GAPDH-as (GAAGATGGTGATGGGATTTC). PCR reactions were set up in duplicate or triplicate and amplified in a LightCycler 480 thermal cycler; ACTB (β-actin), B2M (β-2 microglobulin) or GAPDH was used as an endogenous control. RT-PCR to detect viral transcripts was performed as described elsewhere (Rende et al., 2011). Primers were synthesized by Sigma-Genosys.

### Plasmids and Transfections

Plasmid pGFP-miR-34a-sponge was constructed by inserting the GFP coding sequence followed by four miR-34a target sequences into pcDNA3.1 (Invitrogen); a control plasmid lacking the miR-34a target sequences (GFP-control) was also cloned. The inserts were obtained from previously described retroviral vectors (Rao et al., 2010). In **Figure 4C**, HeLa cells were transfected using PolyJet transfection reagent (SignaGen Laboratories). In **Figure 7**, C91PL cells were electroporated as described (Silic-Benussi et al., 2010). DNA transfection mixtures and incubation times are indicated in the figure legends.

### Drug Treatments

Bay 11-7082 and nutlin-3a (Sigma-Aldrich) were dissolved in dimethyl sulfoxide (DMSO, Hybrimax; Sigma-Aldrich). Cells were seeded in tissue culture plates at 300,000 cells/mL and treated with the drugs or with the same volume of DMSO (final dilution, 0.1%) for 48 h. Nutlin-3a was substituted with nutlin-3 (Tocris Bioscience) in some replicates; no substantial difference was noted in the effects of the two preparations.

### Immunoblotting

Cells were lysed in Cell Disruption Buffer (Ambion) containing inhibitors of proteases and phosphatases (Complete and PhosSTOP, Roche); samples to be analyzed for p53 acetylation were supplemented with 10 mM nicotinamide (Sigma-Aldrich) to inhibit deacetylases. Samples were analyzed with a Bradford protein assay (Bradford, 1976), balanced for total protein and subjected to SDS-PAGE followed by electrotransfer to nitrocellulose membrane (GE Healthcare). Blots were saturated in non-fat milk (Euroclone) and incubated with rabbit anti-GAPDH antibody (Genetex), rabbit anti-β-actin polyclonal antibody (Sigma-Aldrich), rabbit anti-acetylated p53 antibody (Lysine-382, Cell Signaling), goat anti-p53 polyclonal antibody, and rabbit anti-SIRT1 polyclonal antibody (both from Santa Cruz Biotechnology) followed by horseradish peroxidase-conjugated secondary antibodies (Pierce or GE Healthcare). Immunoreactive bands were detected using Femto (Pierce) or LiteAblot Turbo (Euroclone) chemiluminescence reagent and a digital imager (BioRad ChemiDoc XRS or Cambridge UVTEC).

### Analyses of Cell Turnover

For cell cycle analysis, cells were fixed in ethanol (Sigma-Aldrich), stained with propidium iodide (Sigma-Aldrich) in the presence of RNase A (Qiagen) and analyzed by flow cytometry using a FACSCalibur (BD, FL2-A setting) and ModFit software. Uptake of Live-Dead Far Red (Molecular Probes) was measured according to the manufacturer’s instructions. Conversion of 3-(4, 5-dimethylthiazol-3-yl)-2,5-diphenyl tetrazolium bromide (MTT, Sigma-Aldrich) to blue formazan (MTT test; Mosmann, 1983) was measured using a standard protocol.

## Results

### miR-34a Expression Is Increased in HTLV-1-Positive Cell Lines and Samples From ATLL Patients

**FIGURE 1:**
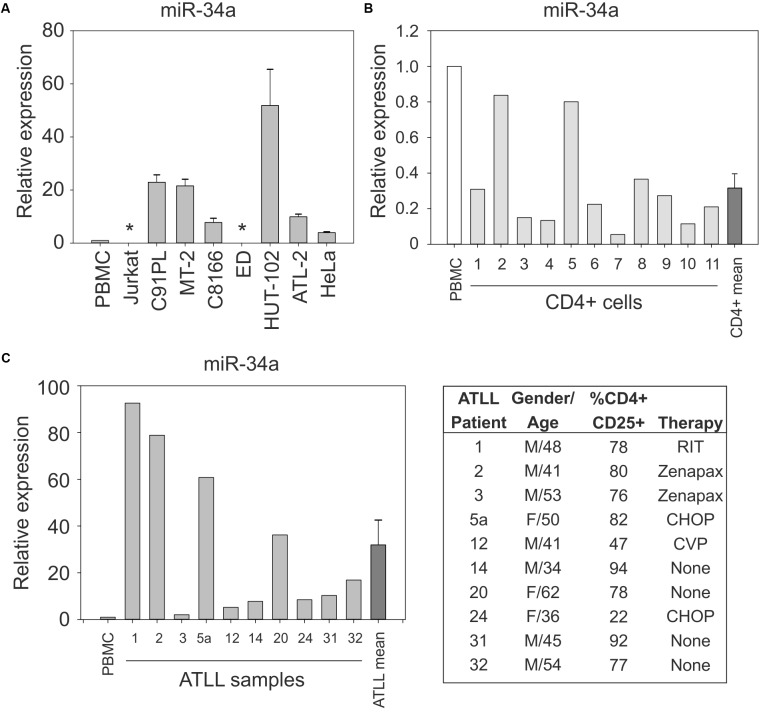
Expression of miR-34a in normal CD4+ cells, HTLV-1-infected cell lines, and ATLL samples. miR-34a was detected by qRT-PCR as described in the Section “Materials and Methods,” with values measured in PBMC samples from healthy donors used as a calibrator (set at 1 in graphs). Panel **(A)** shows miR-34a levels in the indicated cell lines. Bars represent mean values from three to seven samples of cells with standard error. ^∗^miR-34a was below the limit of detection in cell lines ED and Jurkat. Panel **(B)** shows the expression of miR-34a in 11 samples of normal unstimulated CD4+ cells. The mean relative expression value for miR-34a in the CD4+ cell samples was 0.316 (bar labeled CD4+ mean). Panel **(C)** shows expression of miR-34a in 10 ATLL samples. The mean relative expression value for miR-34a in the ATLL samples was 31.91 (bar labeled ATLL mean). The table reports characteristics of the ATLL patients. Patient number 24 had ATLL manifested as a lymphoma. M, male; F, female; RIT, radioimmunotherapy; Zenapax, humanized monoclonal antibody against IL-2Rα; CHOP, cyclophosphamide, doxorubicin, vincristine, and prednisone; CVP, cyclophosphamide, vincristine, and prednisone.

**Figure 1A** shows the results of quantitative RT-PCR (qRT-PCR) to detect miR-34a in normal PBMC samples and in the HTLV-1-positive cell lines C91PL, MT-2, HUT-102, C8166, ATL-2, and ED, and in the uninfected cell lines HeLa and Jurkat; results were scaled against values measured in normal PBMC. These assays revealed that, with the exception of ED cells, all of the HTLV-1-positive cell lines expressed much higher levels of miR-34a compared to PBMC, HeLa, and Jurkat cells. Additional assays carried out on 11 samples of purified CD4+ cells from healthy donors indicated variable levels of miR-34a, which were lower than those measured in unfractionated PBMC (**Figure 1B**).

Results of qRT-PCR on PBMC isolated from 10 ATLL patients (described in **Figure 1C** and Pise-Masison et al., 2009) revealed increased levels of miR-34a in all of the patients’ samples compared to the PBMC calibrator (mean 32-fold increase; **Figure 1C**).

### Analysis of miR-34a Promoter Methylation Status in HTLV-1-Positive Cell Lines

The miR-34a gene is coded on the complementary strand of chromosome 1p36.22, a region that is frequently deleted in cancer (Calin et al., 2004). The miR-34a primary RNA (pri-miRNA) contains two or more exons. The region upstream of exon 1 contains many CpG dinucleotides that can be methylated, an event that contributes to the silencing of miR-34a expression in various tumor-derived cell lines and solid cancers, and in some hematological malignancies (Lodygin et al., 2008; Chim et al., 2010; Craig et al., 2011). The substantial difference in miR-34a levels detected in the cell lines shown in **Figure 1A** led us to investigate the methylation status of the miR-34a promoter by performing methylation-specific PCR (MSP) on a 170-bp segment of the miR-34a promoter region as described (Ng et al., 2014).

**FIGURE 2:**
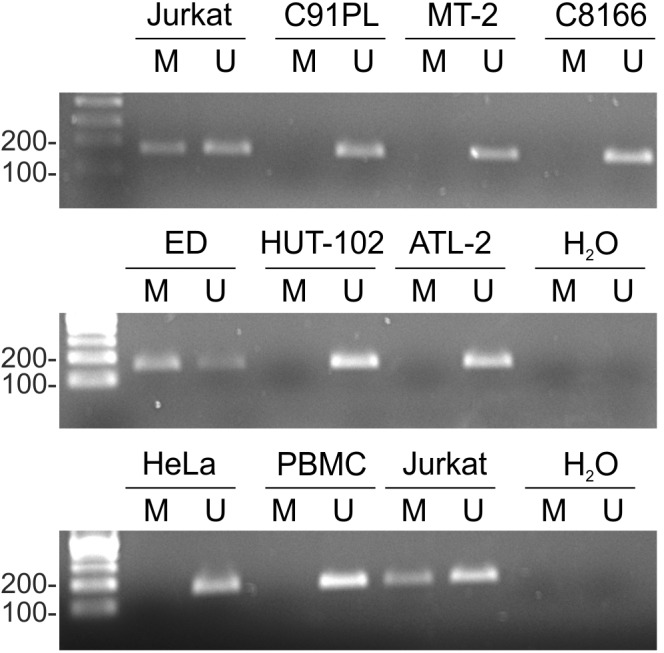
miR-34a promoter methylation in cell lines. Genomic DNA from normal PBMC and the indicated cell lines was subjected to MSP as described in the Section “Materials and Methods.” Shown are resulting PCR products after separation in 2% agarose gels. The first lane in each image contains a 100-bp DNA marker (Sharpmass 100, Euroclone). Identical results were obtained for a second DNA sample of each cell type; Jurkat cells are shown as an example.

Results of MSP showed that the miR-34a promoter was substantially methylated in Jurkat and ED cells, but not in cell lines C91PL, MT-2, HUT-102, C8166, or ATL-2 (**Figure 2**). HeLa cells and normal PBMC also did not yield methylated products. Sequencing analysis of the methylated PCR product obtained for ED cells confirmed efficient bisulfite conversion and revealed methylation of 13 CpGs lying internal to the MSP PCR primers (data not shown).

### miR-34a Is Upregulated in Newly Infected PBMC

**FIGURE 3:**
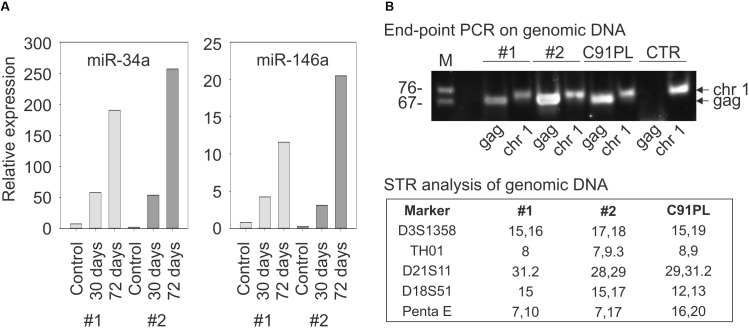
Changes in miR-34a and miR-146a levels upon infection of PBMC. Panel **(A)** shows miR-34a and miR-146a levels in PBMC from two healthy donors (distinguished by light- and dark gray bars) harvested 7 days after stimulation with PHA and IL-2 (Controls) and 30 or 72 days after PHA/IL-2 stimulation and co-cultivation with lethally irradiated C91PL cells. Relative expression values were calculated using the control PBMC as calibrators (set at 1 in the graphs). Panel **(B)** shows results of PCR to detect HTLV-1 sequences (lanes labeled HTLV-1) in the genomic DNA of infected PBMC cultures #1 and #2 at day 72. PCR to detect the pre-miR-34a genomic region (lanes labeled pre-miR-34a) served as a control for amplification of genomic DNA, and genomic DNA from C91PL cells and from PBMC of an uninfected donor (CTR) served as positive and negative controls for amplification of HTLV-1 DNA. The table reports values for selected short tandem repeat markers that distinguished the infected PBMC cultures from input C91PL cells.

To further examine the link between HTLV-1 infection and miR-34a expression, PBMC from two healthy donors were infected with HTLV-1 through co-cultivation with lethally irradiated C91PL cells and then analyzed by qRT-PCR. Results showed a progressive increase in miR-34a levels after 30 and 72 days of culture compared to the uninfected PBMC (left-hand graph, **Figure 3A**). The infected cultures also showed an increase in the levels of miR-146a, a miRNA that is known to be upregulated by Tax-mediated NF-κB stimulation (Pichler et al., 2008; Tomita et al., 2009; right-hand graph, **Figure 3A**). Results of end-point PCR on genomic DNA isolated at day 72 of the experiment using primers specific for the HTLV-1 *gag* gene confirmed that both co-cultures were HTLV-1-infected (**Figure 3B**). Short tandem repeat (STR) analysis yielded distinct profiles for the two co-cultures and the C91PL cell line, thus verifying that the co-cultures no longer contained input C91PL cells (table in **Figure 3B**).

### Identification of a Spliced pri-miR-34a in C91PL Cells

**FIGURE 4:**
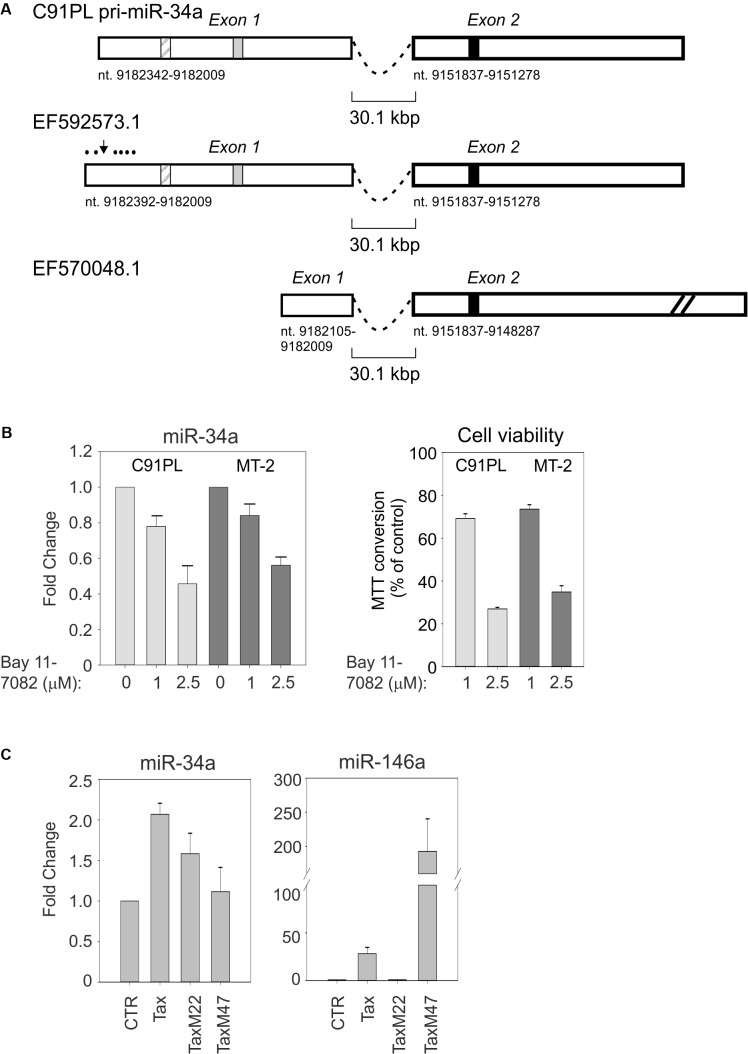
C91PL cells produce a pri-miR-34a transcript that contains binding sites for NF-κB and p53. Panel **(A)** shows the spliced pri-miR-34a identified by RACE in the present study of C91PL cells, in a previous study of HeLa cells (EF592573.1; Chang et al., 2007) and in a lung cancer cell line engineered to produce p53 (EF570048.1; Tarasov et al., 2007). Two additional spliced pri-miR-34a transcripts identified in phorbol ester-treated K562 cells contain 2 exons located 5′ to exon 1 (not shown; Navarro et al., 2009). Numbering is according to the GenBank GRCh38.p12 primary assembly (minus strand). The box with diagonal lines indicates an NF-κB binding site (nt 9182264-9182255; Li et al., 2012), the gray box indicates a p53 binding site (nt 9182163-9182144; Raver-Shapira et al., 2007) and the black box indicates the position of mature miR-34a (nt 9151756-9151735). In EF570048.1, exon 2 is drawn at reduced scale (indicated by diagonal lines). Panel **(B)** shows expression of miR-34a and cell viability in C91PL and MT-2 cells after treatment for 48 h with the indicated concentrations of the NF-κB inhibitor Bay 11-7082; control cultures were treated with the same volume of DMSO (set at 1). Cell viability was measured as MTT conversion. In Panel **(C)**, HeLa cells were transfected with a plasmid coding for wildtype Tax, TaxM22, or TaxM47 (Smith and Greene, 1990) or with pBluescript KS+ (Stratagene; CTR, set at 1) and analyzed for expression of miR-34a and miR-146a. All graphs show mean values from three experimental replicates with standard error bars, scaled against controls.

Alternatively spliced miR-34a pri-miRNAs have been identified in different cell contexts. 5′RACE and 3′RACE on total RNA isolated from C91PL cells followed by sequencing analysis yielded a 2-exon pri-miR-34a of 894 nt (**Figure 4A**). This pri-miRNA is similar to a miR-34a precursor designated EF592573.1 that was previously identified in HeLa cells (**Figure 4A**; Chang et al., 2007). It is noteworthy that exon 1 of this pri-miRNA contains a binding site for NF-κB and a binding site for p53 that were shown to be engaged by these transcription factors in other cell systems (Tarasov et al., 2007; Li et al., 2012). Another miR-34a pri-miRNA designated EF570048.1, which was identified in a lung cancer cell line induced to express p53 (Tarasov et al., 2007), does not contain the p53- and NF-κB binding sites in exon 1 and contains a longer exon 2 sequence (**Figure 4A**).

The presence of an NF-κB binding site in the miR-34a pri-miRNA identified in C91PL cells was of interest, as constitutive activation of the NF-κB pathway is a hallmark of HTLV-1 infection/transformation (reviewed by Sun and Yamaoka, 2005). This led us to test the effects of the NF-κB inhibitor Bay 11-7082 (Pierce et al., 1997) on miR-34a expression in C91PL and MT-2 cells. As shown in **Figure 4B**, treatment with Bay 11-7082 led to a dose-dependent reduction in miR-34a levels in both cell lines, thus suggesting that NF-κB contributes to sustain miR-34a expression. A previous study showed that treatment of HTLV-1-infected cell lines and primary ATLL cells with Bay 11-7082 caused a reduction in the expression of NF-κB-responsive genes, accompanied by reduced cell viability and increased apoptosis (Mori et al., 2002). In line with these findings, we observed a dose-dependent loss of viability in the Bay 11-7082-treated C91PL and MT-2 cultures (**Figure 4B**).

miR-34a was previously observed to be upregulated in the Epstein–Barr virus (EBV)-infected B-cells through LMP1-mediated stimulation of the NF-κB pathway (Forte et al., 2012). The ability of Tax to activate NF-κB, CREB, and other transcription factors led us to test its effects on the expression of miR-34a in HeLa cells transfected with wildtype Tax and Tax mutants defective for activation of NF-κB or CREB. Results showed that miR-34a expression was increased by about twofold by wildtype Tax, and by about 1.5-fold by NF-κB-pathway-defective TaxM22, while TaxM47, defective for CREB activation, did not substantially affect miR-34a levels (**Figure 4C**). In contrast, miR-146a, known to be regulated by NF-κB, was strongly induced by wildtype Tax and TaxM47, but not by TaxM22.

### p53 Activation Enhances miR-34a Expression in HTLV-1-Positive Cell Lines

**FIGURE 5:**
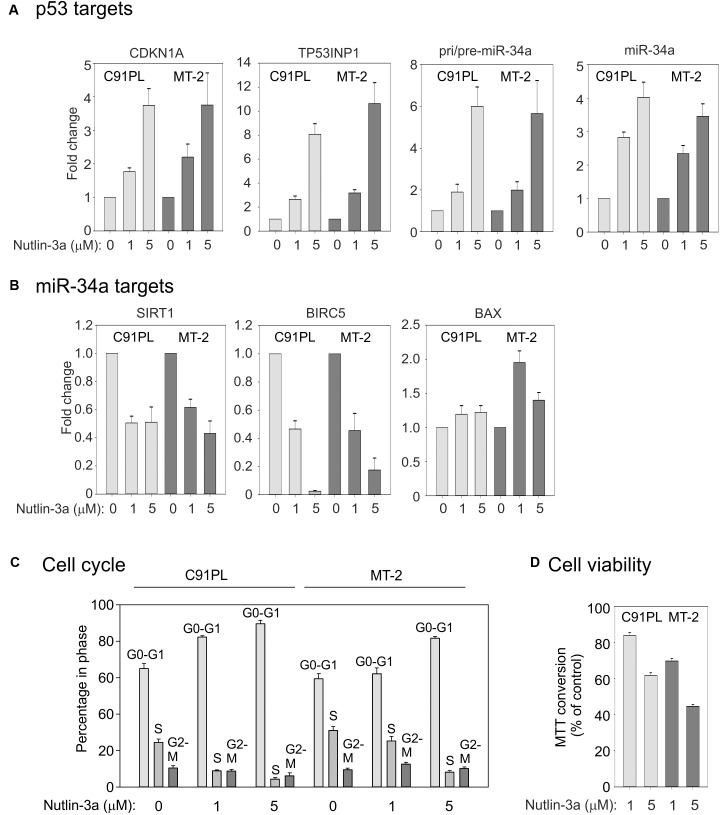
Effects of nutlin-3a in C91PL and MT-2 cells. C91PL and MT-2 cells were treated with 1 or 5 μM nutlin-3a or with the same volume of DMSO for 48 h and then analyzed for expression of p53-responsive mRNAs, including pri-/pre-miR-34a and mature miR-34a [three experiments, Panel **(A)**] and miR-34a target transcripts [three experiments, Panel **(B)**]. Relative expression values were scaled against DMSO-treated controls (set at 1). Panel **(C)** shows cell cycle analysis measured in three experiments. Panel **(D)** shows cell viability measured by MTT conversion (six experiments). Graphs show mean values with standard error.

We next investigated the effects of activation of p53 on miR-34a expression in C91PL and MT-2 cells, which were reported to produce wildtype p53 that is however functionally defective (Cereseto et al., 1996; Kamihira et al., 2001; Hasegawa et al., 2009). To activate p53 we treated the cell lines with nutlin-3a, which stabilizes p53 through inhibition of MDM2 (Vassilev et al., 2004). Results of qRT-PCR showed that nutlin-3a treatment resulted in increased expression of the p53 target genes CDKN1A (coding for p21Waf1/Cip1), TP53INP1, pri/pre-miR-34a, and mature miR-34a (**Figure 5A**).

qRT-PCR analysis to compare the expression of 12 other miRNAs in C91PL cells (Supplementary Figure 1) revealed that most of the tested miRNAs were downregulated by nutlin-3a, including miR-93 and miR-130b, whose levels are elevated in HTLV-1-positive cell lines and ATLL samples (Yeung et al., 2008). Of note, miR-125b, which is known to repress p53 expression in neuroblastoma cells and fibroblasts (Le et al., 2011), was upregulated with nutlin-3a treatment (Supplementary Figure 1).

We next investigated the expression of miR-34a target mRNAs coding for the NAD^+^-dependent protein deacetylase SIRT1 (Yamakuchi et al., 2008), the inhibitor of apoptosis (IAP) family member BIRC5 (Survivin; Chen et al., 2010; Kaller et al., 2011; Shen et al., 2012) and the pro-apoptotic protein BAX (Fan and Wang, 2016). qRT-PCR results demonstrated a ∼50% reduction in the SIRT1 mRNA, a more substantial reduction in BIRC5, and a marginal increase in the BAX mRNA (**Figure 5B**). The strong reduction in BIRC5 was likely due to the combined effect of miR-34a and transcriptional repression p53 (Hoffman et al., 2002), while the modest increase in BAX could be explained by the fact that BAX is both a target for repression by miR-34a and for transcriptional upregulation by p53 (Miyashita et al., 1994). Consistent with previous studies (Hasegawa et al., 2009) and with its effects on CDKN1A expression, nutlin-3a caused a block of C91PL and MT-2 cells in G0/G1 (**Figure 5C**), a reduction in viability (measured using the MTT assay **Figure 5D**), and increased staining with annexin V, a marker of apoptosis (data not shown). Increased levels of CDKN1A and miR-34a and a reduction in the levels of SIRT1 and cell viability were also observed in nutlin-3a-treated HUT-102 cells (Supplementary Figure 2). Results of cDNA sequencing confirmed that cell lines C91PL, MT-2, and HUT-102 coded for wildtype p53 protein (data not shown).

### Engagement of the miR-34a-p53 Feedback Loop in Nutlin-3a-Treated Cells

**FIGURE 6:**
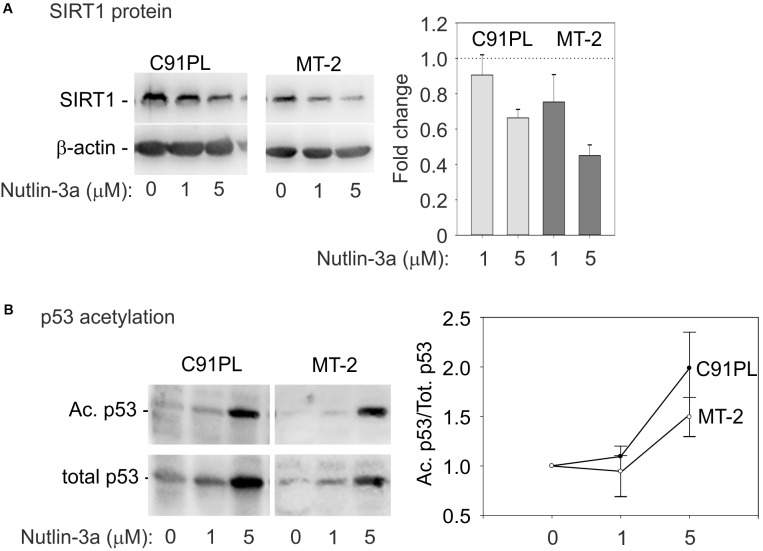
Activation of the p53-miR-34a-SIRT1 feedback loop in C91PL and MT-2 cells. Panel **(A)** shows composite images of immunoblots to detect SIRT1 protein in lysates of control and nutlin-3a-treated cells, with β-actin serving as a loading control. The graph shows the mean fold change in SIRT1 protein expression normalized against β-actin measured in 3 experiments with standard error bars. In Panel **(B)**, lysates were immunoblotted with an antibody specific for p53 acetylated on lysine 382. The blots were then stripped and incubated with an anti-p53 antibody. Signal intensities were used to calculate ratios of acetylated p53 to total p53 in each sample. Values were scaled against the ratio calculated for the untreated cells to express a fold-change of acetylated/total p53 in the treated samples compared to untreated controls. The plot shows mean scaled ratios measured in three experiments with standard error bars.

Substrates of SIRT1 include lysine 382 of p53 (Nakamura et al., 2000; reviewed by Martinez-Redondo and Vaquero, 2013), whose deacetylation interferes with the tumor suppressor’s functional activity (reviewed by Reed and Quelle, 2014). We therefore measured the effects of nutlin-3a on the levels of SIRT1 protein, total p53, and lysine 382-acetylated p53. Results showed that nutlin-3a treatment produced a reduction in the levels of SIRT1 (**Figure 6A**), and an increase in total p53 and acetylated p53 (**Figure 6B**), with a relative increase in acetylation on lysine 382 (graph, **Figure 6B**). This observation indicated that nutlin-3a engages the p53-miR-34a-SIRT1 positive feedback loop in infected cells.

### Effects of an miR-34a Mimic and Sponge in C91PL Cells

**FIGURE 7:**
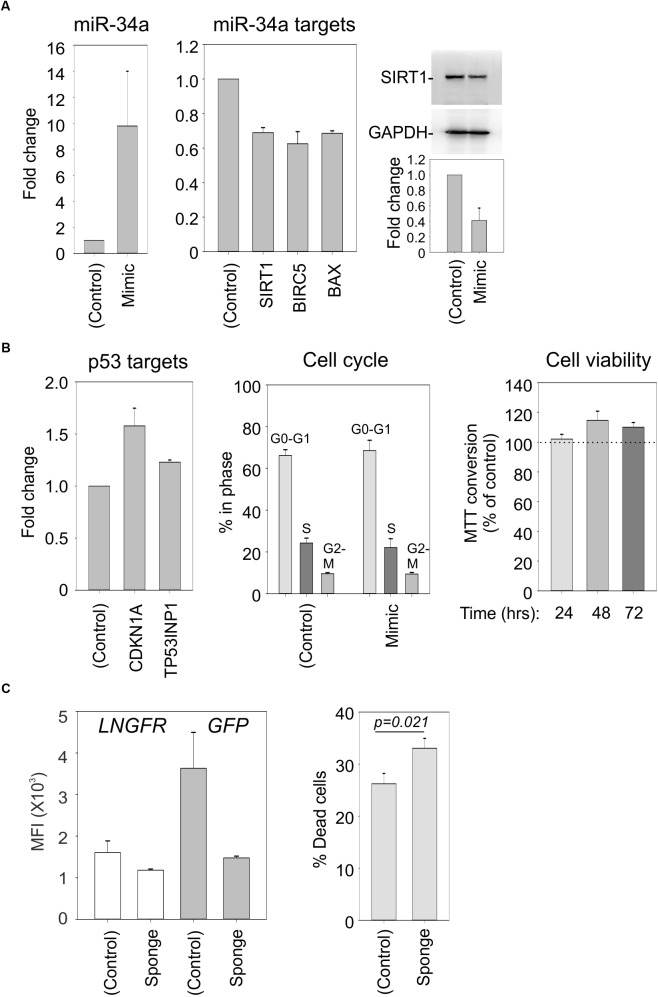
Effects of a miR-34a mimic and sponge in C91PL cells. In Panels **(A)** and **(B)**, C91PL cells (4 × 10^6^ cells, 4 μg DNA in 100 μL Buffer R) were electroporated with a miR-34a-mimic or a control synthetic RNA and analyzed 72 h later for changes in miR-34a and the indicated mRNAs (3–6 experimental replicates), SIRT1 protein (four replicates), and cell cycle (five replicates). Cell viability measured with the MTT test (four replicates) at the indicated time points after electroporation was scaled against values obtained for control-electroporated cultures at the corresponding time point. Graphs show mean values and standard error bars. The immunoblots show a representative experiment to quantify the change in SIRT1 normalized against GAPDH protein. In Panel **(C)**, C91PL cells were electroporated with pGFP-control or pGFP-miR-34a-sponge (see section “Materials and Methods”) together with pMACS-LNGFR (Miltenyi Biotec, coding for truncated nerve growth factor receptor) and pBluescript KS+ (included as carrier). Three days later, aliquots of cells were labeled with APC-conjugated anti-LNGFR antibody (Miltenyi Biotec) or with Live/Dead Far Red (Invitrogen) and FITC-conjugated anti-LNGFR antibody (Miltenyi Biotec) and analyzed with a BD FACSalibur. The left-hand plot shows the mean fluorescence intensities (MFI) measured for the LNGFR and GFP signals in the GFP+LNGFR double-positive populations from three transfections with standard error bars. The right-hand plot shows mean % cell death values [=(% of Live/Dead Far Red+LNGFR-labeled cells/total % LNGFR-positive cells) × 100] from 11 transfections with standard error bars (*p* = 0.021, Mann–Whitney rank sum test).

To verify that SIRT1, BIRC5, and BAX represent direct targets of miR-34a in our cell system, we electroporated C91PL cells with a synthetic miR-34a mimic or control RNA. Results of RT-PCR revealed that the mimic-transfected cells had increased levels of miR-34a and reduced levels of the SIRT1, BIRC5, and BAX mRNAs (**Figure 7A**), thus providing direct evidence for targeting of these mRNAs by miR-34a. Immunoblot analysis confirmed the downregulation of SIRT1 protein in the miR-34 mimic-transfected cells (**Figure 7A**, right panel). However, the miR-34a mimic did not have a substantial effect on the levels of mRNAs coding for the p53 targets CDKN1A and TP53INP1, nor did it affect the cell cycle profile or cell viability (**Figure 7B**).

Studies of miR-34a in EBV-infected cells showed that its functional knockdown results in increased cell death (Forte et al., 2012). In an analogous experiment, we electroporated C91PL cells with a GFP expression plasmid containing four binding sites for miR-34a in its 3′UTR (“GFP-sponge”) or with a control GFP plasmid lacking the miR-34a binding sites; all transfections included a plasmid expressing LNGFR as a transfection standard. Results of flow cytometry analyses after 3 days of culture showed that while the control- and sponge-transfected cells expressed similar levels of LNGFR, the expression of the GFP-miR-34a sponge was considerably lower than that of the GFP control (**Figure 7C**, left panel), an indication that endogenous miR-34a was silencing the sponge construct. An analysis of Live/Dead Far Red staining revealed an increase in death in the cultures transfected with the miR-34a-sponge compared to cells transfected with the control plasmid (**Figure 7C**, right panel). These observations provide evidence that, in analogy to observations made in EBV-infected B-cells, miR-34a favors the survival of C91PL cells.

## Discussion

Accumulated data indicate that HTLV-1 infection has an important impact on the pattern of microRNA expression in the host cell (reviewed by Moles and Nicot, 2015). In the present study we provide evidence indicating that HTLV-1 infection results in a substantial upregulation of miR-34a expression (**Figure 3**) that is sustained in most HTLV-1-positive cell lines (**Figure 1A**). Interestingly, miR-34a is also elevated in primary PBMC samples from ATLL patients (**Figure 1C**), suggesting that high miR-34a levels provide a selective advantage to HTLV-1-infected cells *in vivo* that persists during the complex process of transformation.

The hypothesis that, rather than representing a functionally irrelevant side effect of infection, upregulation of miR-34a contributes a pro-survival advantage to HTLV-1-infected cells is supported by the observation that its functional knockdown resulted in an increase in death of C91PL cells (**Figure 7C**) and is in line with observations made in EBV-infected cells (Forte et al., 2012).

The presence of an NF-κB binding site in the spliced pri-miR-34a identified in C91PL cells (**Figure 4A**) along with the observation that pharmacological inhibition of NF-κB with Bay 11-7082 resulted in a reduction of miR-34a expression in C91PL and MT-2 cells (**Figure 4B**) suggested that this pathway contributes to sustain miR-34a levels in HTLV-1-infected cells. However, we cannot exclude the possibility that the effect of Bay 11-7082 on miR-34a levels in part reflected a general inhibition of gene expression that accompanied the substantial reduction in cell viability (**Figure 4B**).

We considered Tax to be a likely candidate for activating miR-34a, given its ability to activate the NF-κB pathway. It was therefore surprising that both wildtype Tax and Tax defective for NF-κB activation (TaxM22) produced a modest increase in miR-34a in HeLa cells, while the CREB pathway-defective mutant (TaxM47) did not induce miR-34a (**Figure 4C**). While this result may reflect limitations of this cell line as an experimental system for studying miR-34a regulation, we must also consider the possibility that Tax affects miR-34a through its interactions with CREB (reviewed by Nyborg et al., 2010) or with other transcription factor complexes such as AP-1 (reviewed by Gazon et al., 2017). Furthermore, other viral and cellular factors besides Tax are likely to play a role in miR-34a expression. This latter possibility is supported by the fact that ATLL cells express little or no Tax (Takeda et al., 2004; Yamagishi et al., 2012). In contrast, ATLL cells consistently express HBZ (Satou and Matsuoka, 2007). Results of qRT-PCR assays on five of the ATLL samples analyzed in the present study (nos. 1, 3, 14, 20, 31) confirmed the presence of HBZ mRNA in all of the samples, while Tax/Rex mRNA was undetectable (Supplementary Figures 3A,B). The levels of HBZ mRNA in these samples did not appear to correlate with their differences in miR-34a expression (**Figure 1C**).

Our experiments with nutlin-3a confirmed that miR-34a is a transcriptional target of p53 in C91PL, MT-2, and Hut-102 cells (**Figure 5A** and Supplementary Figure 2), and provided evidence that, by repressing SIRT1, miR-34a reinforces p53 activation (**Figures 5B**, **6**). In contrast, treatment of ED cells with nutlin-3a did not result in an increase in p53 protein, and miR-34a remained undetectable (data not shown). This cell line, which was derived from leukemic cells of an ATLL patient, is defective for p53 expression (Ju et al., 2014) and contains a premature stop codon in the Tax open reading frame (Takeda et al., 2004). An analysis of ATLL sample nos. 1, 3, 14, 20, 31 for p53 mRNA revealed varying levels of expression (Supplementary Figure 3C) that did not appear to correlate with levels of miR-34a (**Figure 1C**).

Following the description of miR-34a as a transcriptional target of p53 (Bommer et al., 2007; Chang et al., 2007; He et al., 2007; Raver-Shapira et al., 2007; Tazawa et al., 2007), studies of the impact of p53 on the miRNA regulatory network identified many miRNAs whose expression is increased due to p53-mediated transcriptional upregulation or through p53-enhanced processing of miRNA precursors (reviewed by Hermeking, 2012). miR-145, a miRNA that is upregulated by p53 through its effects on Drosha-mediated pri-miRNA processing (Suzuki et al., 2009), is of interest, given its downregulation in the context of HTLV-1 and ATLL (Xia et al., 2014). Results of qRT-PCR experiments indicated that miR-145 is not expressed in untreated or nutlin-3a-treated C91PL and HUT-102 cells (data not shown). miR-107, which is upregulated by p53 and regulates hypoxic signaling in the colon cancer cell line HCT116 (Yamakuchi et al., 2010), showed a slight reduction in nutlin-3a-treated C91PL cells (Supplementary Figure 1). The lack of an increase in the levels of miR-145 and miR-107 upon nutlin-3a treatment suggests that p53 status might not be a major determinant controlling expression of these miRNAs in the context of HTLV-1-infected cells.

In addition to NF-κB and p53, other transcription factors including the p53 family member TAp73 (Agostini et al., 2011), ELK1 (Christoffersen et al., 2010), and transcription factors induced by phorbol esters (Navarro et al., 2009) are capable of upregulating miR-34a expression in different cell contexts (reviewed by Slabakova et al., 2017). The possible impact of these factors in HTLV-1-associated upregulation of miR-34a merits further investigation.

Results of early studies of miR-34a demonstrated that its ectopic expression induced cell cycle arrest or apoptosis in many cell lines (Chang et al., 2007; He et al., 2007; Raver-Shapira et al., 2007; Tarasov et al., 2007; Welch et al., 2007). These and other studies prompted the development of strategies to employ miR-34a to treat cancer (Beg et al., 2017; reviewed by Saito et al., 2015). Electroporation of a miR-34a mimic in C91PL cells led to a reduction in the expression of SIRT1, BIRC5 (Survivin), and BAX, thus providing evidence for a direct role for miR-34a in fine-tuning these targets in the context of HTLV-1 infection (**Figure 7A**). However, in our experiments the synthetic miR-34a mimic was not able to rescue p53 function or reduce cell viability (**Figure 7B**).

Studies of miR-34a have placed emphasis on its many targets that have an impact on cell proliferation and survival, such as MYC (Christoffersen et al., 2010) and MYCN (Cole et al., 2008), MET (He et al., 2007; Li et al., 2009), CCND1 and CDK6 (Sun et al., 2008), BCL2 (Cole et al., 2008), and NOTCH1 (Ji et al., 2008). The possibility that miR-34a favors survival of HTLV-1-infected cells by modulating expression of BAX, a known tumor suppressor (Yin et al., 1997), merits further investigation.

It is noteworthy that SIRT1 is upregulated in HTLV-1-transformed cells, and particularly in acute ATLL (Kozako et al., 2012). Experiments carried out in chronically infected cell lines and ATLL cells showed that siRNA-mediated knockdown of SIRT1 expression or treatment with sirtuin inhibitors results in apoptotic death, suggesting that SIRT1 is important for the survival of HTLV-1-transformed cells (Kozako et al., 2015; Tang et al., 2015). On the other hand, another study indicated that SIRT1 interferes with the ability of Tax to transactivate the LTR promoter (Tang et al., 2015). It is thus possible that, by modulating SIRT1 expression, miR-34a might enhance transcription of the viral genome. Along these lines, it is interesting to note that nutlin-3a substantially increased the levels of viral mRNAs in C91PL and MT-2 cells (Supplementary Figure 4A) and in HUT-102 cells (Supplementary Figure 2B). However, such an increase was not observed upon electroporation of the miR-34a mimic into C91PL cells (Supplementary Figure 4B). A thorough understanding of the impact of miR-34a on HTLV-1-infected T-cells is worthy of further study, given the current interest in the use of miR-34a mimics, nutlin-3a analogs, and SIRT1 inhibitors to treat various cancers.

## Author Contributions

VC and DMD designed the study. CP-M designed the experiments and provided the ATL samples. VKS, VR, KR, IC, and MS-B performed the experiments. All authors interpreted the data and prepared the manuscript.

## Conflict of Interest Statement

The authors declare that the research was conducted in the absence of any commercial or financial relationships that could be construed as a potential conflict of interest.
