# Supplementary material for: Expression of miR-34a in T-Cells Infected by Human T-Lymphotropic Virus 1
Source: Front Microbiol. 2018 May 4;9:832. doi: 10.3389/fmicb.2018.00832 (PMC5945834; doi:10.3389/fmicb.2018.00832)
Supplement: Supplementary file 1 [file Data_Sheet_1.PDF]

Supplemental Figure legends

Supplemental Figure 1. Effects of Nutlin-3a on expression of miRNAs in C91PL cells. Shown are the fold changes in expression of the indicated miRNAs measured in C91PL cells after 48 hrs' treatment with 5 μM nutlin-3a scaled against DMSO-treated controls (means from 3 experiments with standard error).

Supplemental Figure 2. Effects of nutlin-3a in HUT-102 cells. Hut-102 cells were treated with 1- or 5 μM nutlin-3a or with the same volume of DMSO for 48 hrs. Panels A and B show analyses for expression of p53-responsive mRNAs CDKN1A and miR-34a (Panel A) and the indicated viral mRNAs (Panel B). Panel C shows a composite image of an immunoblot to detect SIRT1, p53 and GAPDH in 3 replicate experiments (a,b,c) The lane marked M contained a pre-stained protein marker (Sharpmass VI, Euroclone). Graphs beside the blots show the fold change in p53 and SIRT1 proteins after normalization against GAPDH protein. Panel D shows cell viability measured by MTT conversion after 48 hrs of treatment. All graphs show mean values with standard error from 3 experiments, scaled against untreated samples (set at 1).

Supplemental Figure 3. Levels of viral mRNAs and p53 mRNA in infected cell lines and ATLL samples. Panels A and B show results of qRT-PCR to detect HBZ SP1 mRNA and Tax/Rex mRNA, respectively in the indicated cell lines and ATLL samples (means of duplicate assays). The Tax/Rex mRNA was undetectable (ND) in the ATLL samples. Panel C shows results of qRT-PCR to measure p53 mRNA levels in 3 normal PBMC samples and in the indicated cell lines and ATLL samples (means of duplicate assays) scaled against the mean obtained for the PBMC samples (set at 1).

Supplemental Figure 4. Effects of Nutlin-3a and a miR-34a mimic on expression of viral mRNAs. Panel A shows the fold change in expression of the indicated viral mRNAs measured in C91PL and MT-2 cells after 48 hrs' treatment with 1- or 5 μM nutlin-3a, scaled against DMSO-treated controls.

Graphs show means from 3 experiments with standard error. Panel B shows changes in levels of the viral mRNAs as indicated in Panel A in C91PL cells after transfection with a miR-34a mimic. Shown are results from 6 experiments with standard error, scaled against values measured in cells transfected with control RNA (set at 1 and indicated with dotted line). The Tax/Rex mRNA showed a statistically significant decrease in levels in mimic-transfected cells ($p= 0.002$, Mann-Whitney Rank Sum test).

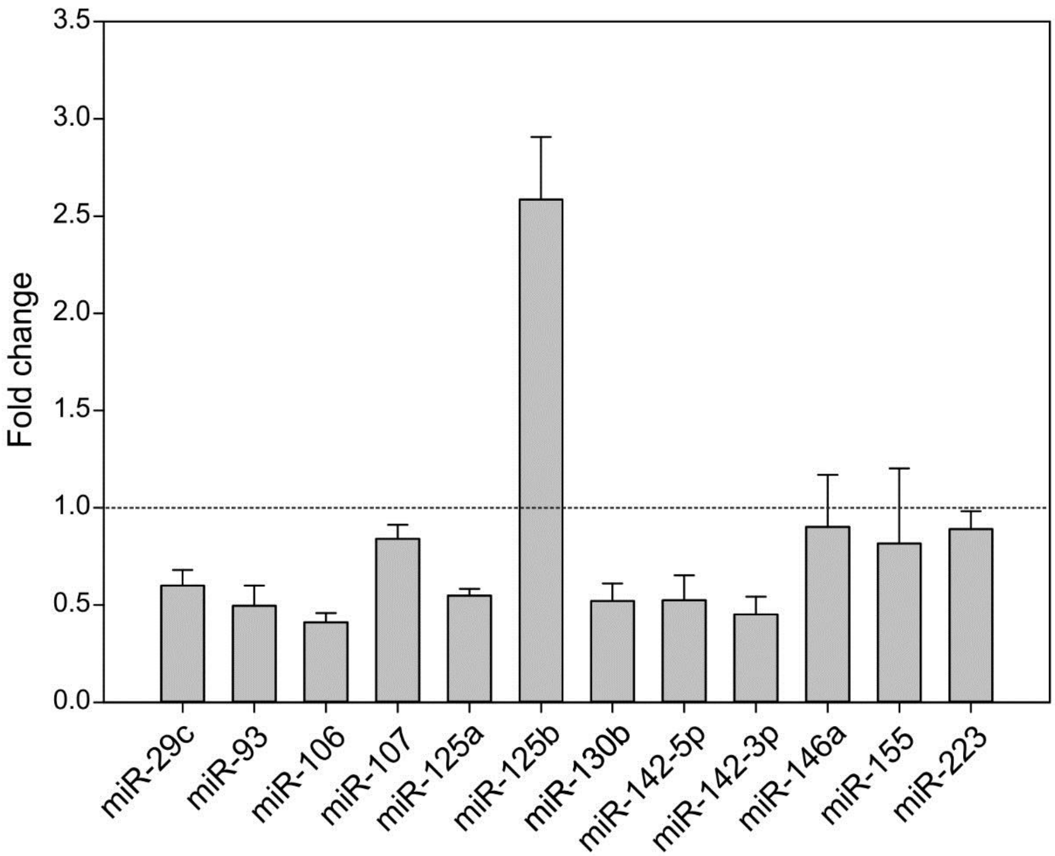

Supplemental Figure 1

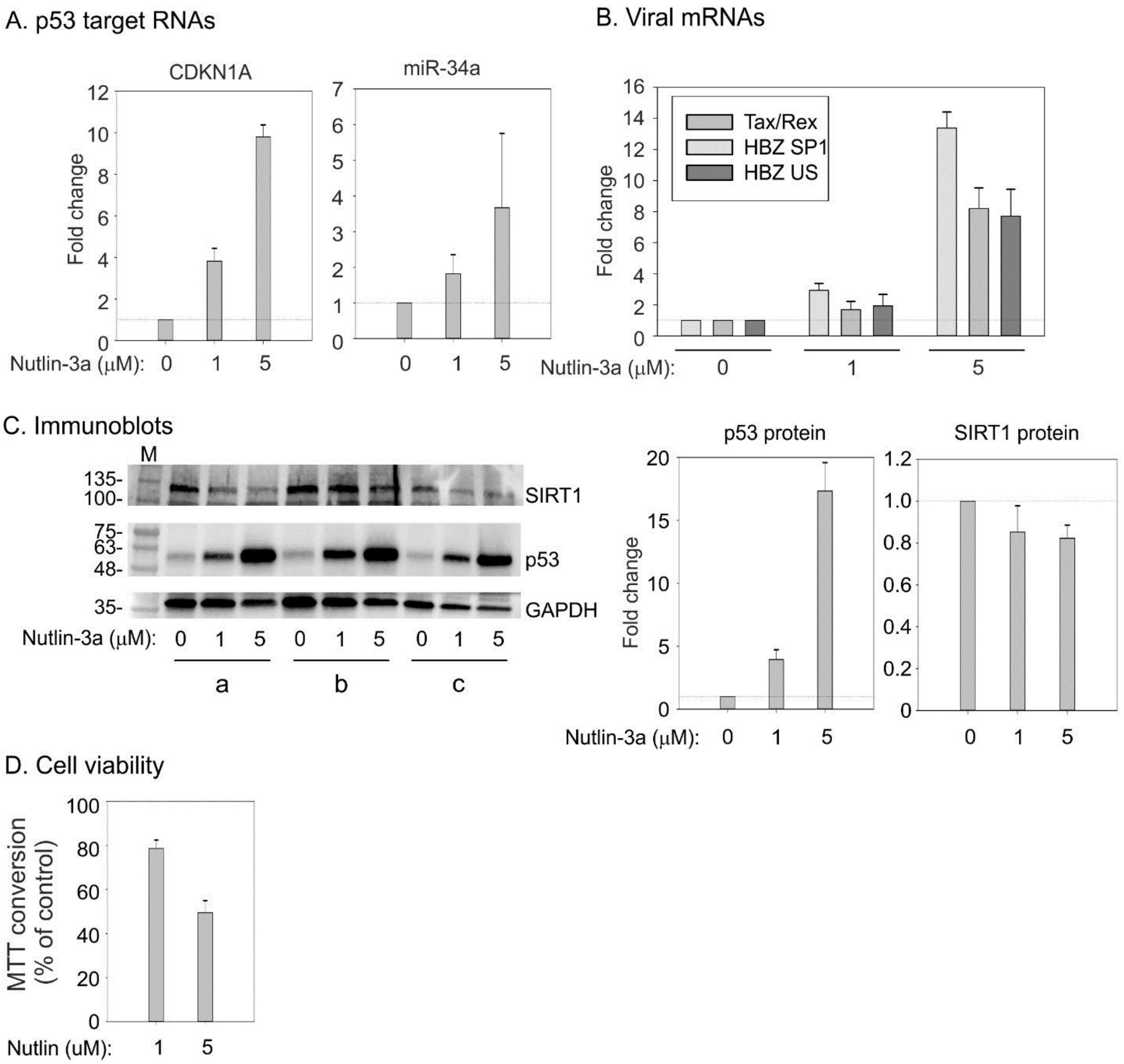

Supplemental Figure 2

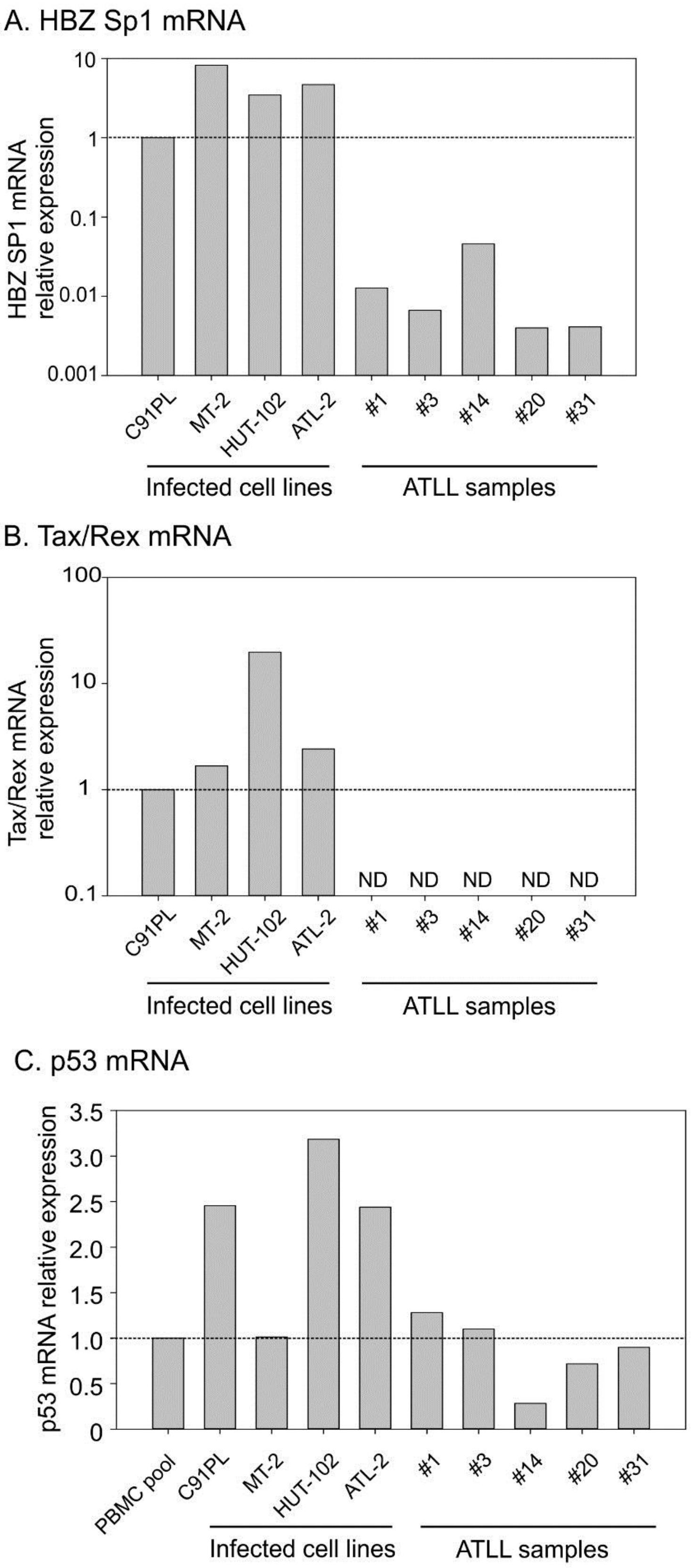

Supplemental Figure 3

A.

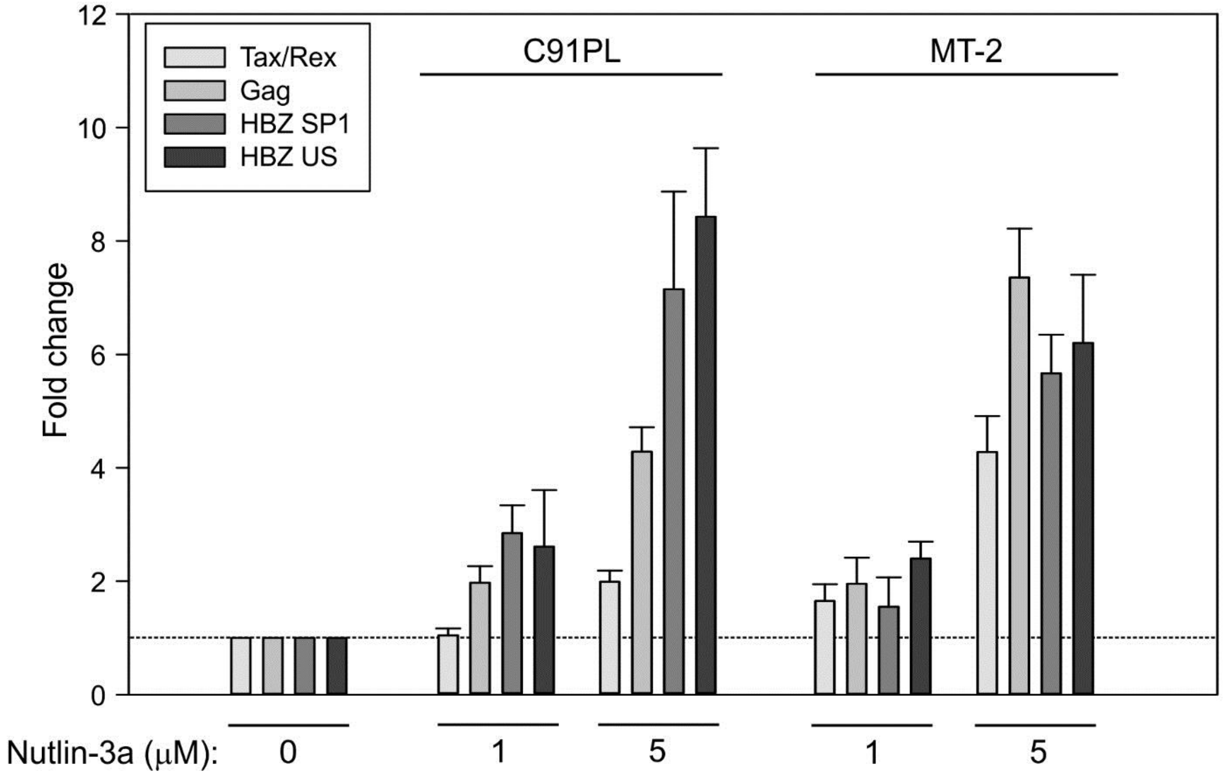

B.

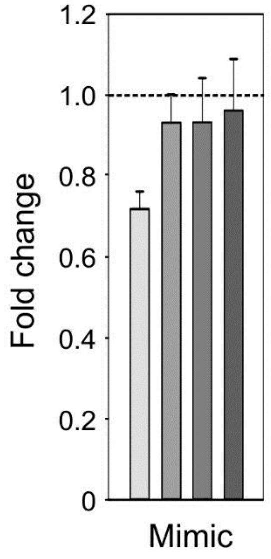

Supplemental Figure 4
